# Supplementary material for: Cisplatin Dependent Secretion of Immunomodulatory High Mobility Group Box 1 (HMGB1) Protein from Lung Cancer Cells
Source: Biomolecules. 2023 Aug 31;13(9):1335. doi: 10.3390/biom13091335 (PMC10526420; doi:10.3390/biom13091335)
Supplement: Supplementary file 1 [file biomolecules-13-01335-s001.zip › biomolecules-2527825-Figure S1-S12.pdf]

Table of Contents

Table S1. Lysine-containing peptides in HMGB1 analyzed by PRM/HRMS.

Figure S1. NanoLC-PRM/HRMS chromatograms of non-cysteine tryptic peptides from cisplatin-treated A549 media.

Figure S2. Calibration curves with HMGB1 tryptic peptide transitions constructed in cell culture media.

Figure S3. HMGB1 and HMGB2 in nuclear, cytosolic, and media fractions of A549 cells.

Figure S4. HMGB1 and HMGB2 in nuclear fraction of cisplatin treated A549 cells.

Figure S5. HMGB1 and HMGB2 in cytosolic fraction of cisplatin treated A549 cells.

Figure S6. HMGB1 and HMGB2 in media from cisplatin treated A549 cells.

Figure S7. NLS1 and NLS2 transitions of unacetylated HMGB1 secreted with cisplatin treatment.

Figure S8. DMSO induces secretion of unacetylated HMGB1.

Figure S9. Assigned mass spectra in cysteine-containing peptides in HMGB1 secreted with cisplatin treatment I.

Figure S10. Assigned mass spectra in cysteine-containing peptides in HMGB1 secreted with cisplatin treatment II.

Figure S11. Cisplatin-mediated secretion of HMGB1 fromA549 cells is not dependent on necrosis.

Figure S12. Cisplatin attenuates A549 cell growth and decreases viability more than other platinum drugs.

Figure S13. Original full size western blots.

Peptide	Protease	Light *m/z*	Heavy *m/z*	Endogenous Acetylation *m/z*				
				5 Ac	4 Ac	3 Ac	2 Ac	1 Ac
GK^{3}GDP$K^{7}K^{8}$PRGK^{12}M(O)SSY	Chymotrypsin	611.3302	625.3582	NA	607.3051	608.3114	609.3176	610.3239
H$K^{28}K^{29}K^{30}$HPDASVNFSE	GluC	586.9755	594.9897	NA	NA	583.9567	584.963	585.9692
FS$K^{43}K^{44}$C(CAM)SE	GluC	488.2398	496.254	NA	NA	NA	485.2209	486.7304
FS$K^{43}K^{44}$C(NEM)SE	GluC	522.2529	530.2671	NA	NA	NA	519.2341	520.7435
K^{57}GK^{59}FE	GluC	698.399	714.4274	NA	NA	NA	692.3614	695.3802
DMAK^{65}ADK^{68}ARYERE	GluC	558.2792	566.9644	NA	NA	NA	556.2666	557.2729
EDMAK^{65}ADK^{68}ARY	Chymotrypsin	694.3433	707.3711	NA	NA	NA	691.3245	692.8339
EREMK^{76}TY	Chymotrypsin	501.2436	510.2643	NA	NA	NA	NA	499.7342
IPPK^{82}GET$K^{86}K^{87}K^{88}$F	Chymotrypsin	726.9461	742.9745	NA	720.9085	722.4179	723.9273	725.4367
K^{90}DPNAPK^{96}RPPSAF	Chymotrypsin	757.9153	765.9295	NA	NA	NA	754.8964	756.4059
DVA$K^{127}K^{128}$LGEMWNNTAA	AspN	869.441	877.4552	NA	NA	NA	866.4222	867.9316
DDK^{141}QPYE$K^{146}K^{147}$AAK^{150}LK^{152}	AspN	629.6945	646.3939	624.6632	625.6694	626.6757	627.682	628.6883
$K^{146}K^{147}$AAK^{150}LK^{152}E	GluC	548.3617	564.3901	NA	542.3241	543.8335	545.3429	546.8523
DIAAYRAK^{165}GK^{167}	AspN	591.841	604.8689	NA	NA	NA	588.8222	590.3316
K^{180}S$K^{182}K^{183}K^{184}K^{185}$E	GluC	550.8608	570.8963	543.3137	544.8231	546.3325	547.8419	549.3513

Table S1. Lysine-containing peptides in HMGB1 analyzed by PRM/HRMS. Coverage of 34 of the 43 lysine residues in HMGB1 (79% coverage) revealed no endogenous acetylation of HMGB1 secreted by cisplatin treated A549 cells. Lysine residues are labeled in red and numbered according to HMGB1 sequence. The m/z for each fully C*D*3-acetylated endogenous HMGB1 peptide and C*D*3-acetylated SILAC HMGB1 peptide are labeled in the Light *m/z* and Heavy *m/z* columns, respectively. The m/z for other possible CH3-acetylated peptides are listed in blue where applicable, with NA signifying that a peptide contains fewer sites of possible acetylation than specified by a given column.

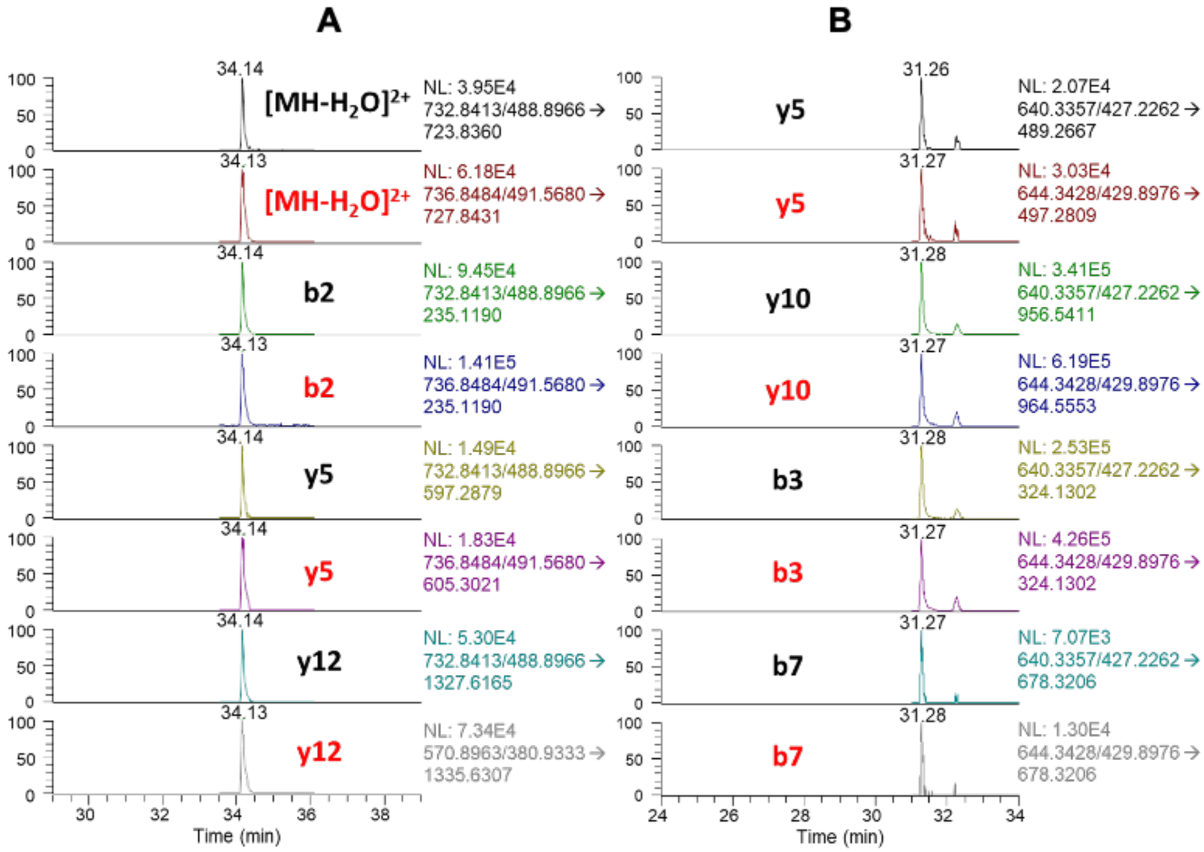

Figure S1. NanoLC-PRM/HRMS chromatograms of non-cysteine tryptic peptides from cisplatin-treated A549 media. Chromatograms are labeled as either light (black) or heavy (red) transitions. **(A)** $H^{31}PDASVNFSEFSK^{43}$ chromatograms. **(B)** $G^{115}EHPGLSIGDVAK^{127}$ chromatograms.

A

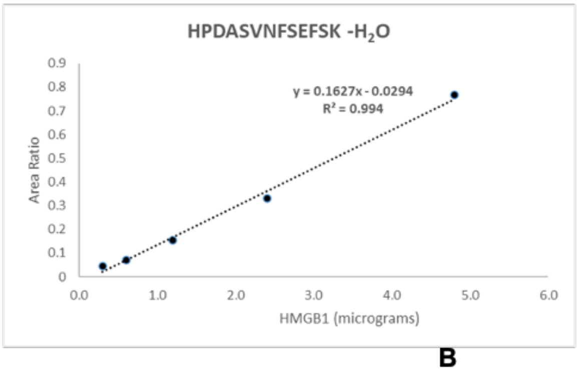

HMGB1 (µg)	Area Ratio	Calculated Value (µg)	Accuracy
0.3	0.045	0.455	51.7%
0.6	0.071	0.615	2.6%
1.2	0.153	1.119	6.7%
2.4	0.329	2.204	8.1%
4.8	0.769	4.906	2.2%

B

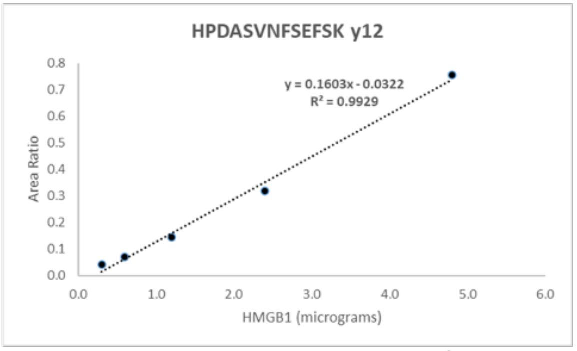

HMGB1 (µg)	Area Ratio	Calculated Value (µg)	Accuracy
0.3	0.042	0.460	53.5%
0.6	0.069	0.634	5.7%
1.2	0.145	1.103	8.1%
2.4	0.318	2.186	8.9%
4.8	0.756	4.917	2.4%

C

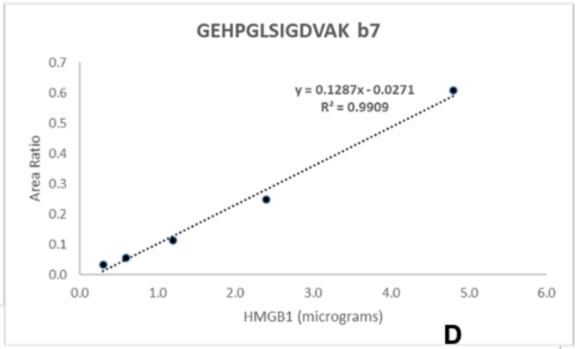

HMGB1 (µg)	Area Ratio	Calculated Value (µg)	Accuracy
0.3	0.034	0.477	59.1%
0.6	0.055	0.639	6.4%
1.2	0.114	1.099	8.4%
2.4	0.250	2.151	10.4%
4.8	0.608	4.932	2.8%

D

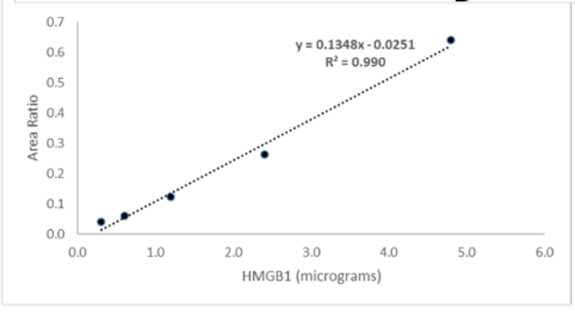

HMGB1 (µg)	Area Ratio	Calculated Value (µg)	Accuracy
0.3	0.040	0.485	61.8%
0.6	0.060	0.632	5.4%
1.2	0.124	1.109	7.6%
2.4	0.262	2.132	11.2%
4.8	0.641	4.940	2.9%

Figure S2. Calibration curves with HMGB1 tryptic peptide transitions constructed in cell culture media. Calculated from the mean of three calibration curves. Accuracy for each calibration curve was calculated with respect to linear regression from the curve. **(A***)* $H^{31}PDASVNFSEFSK^{43}$ dehydration. **(B***)* $H^{31}PDASVNFSEFSK^{43}$ y12**. (C)** $G^{115}EHPGLSIGDVAK^{127}$ b7**. (D)** $G^{115}EHPGLSIGDVAK^{127}$ y10.

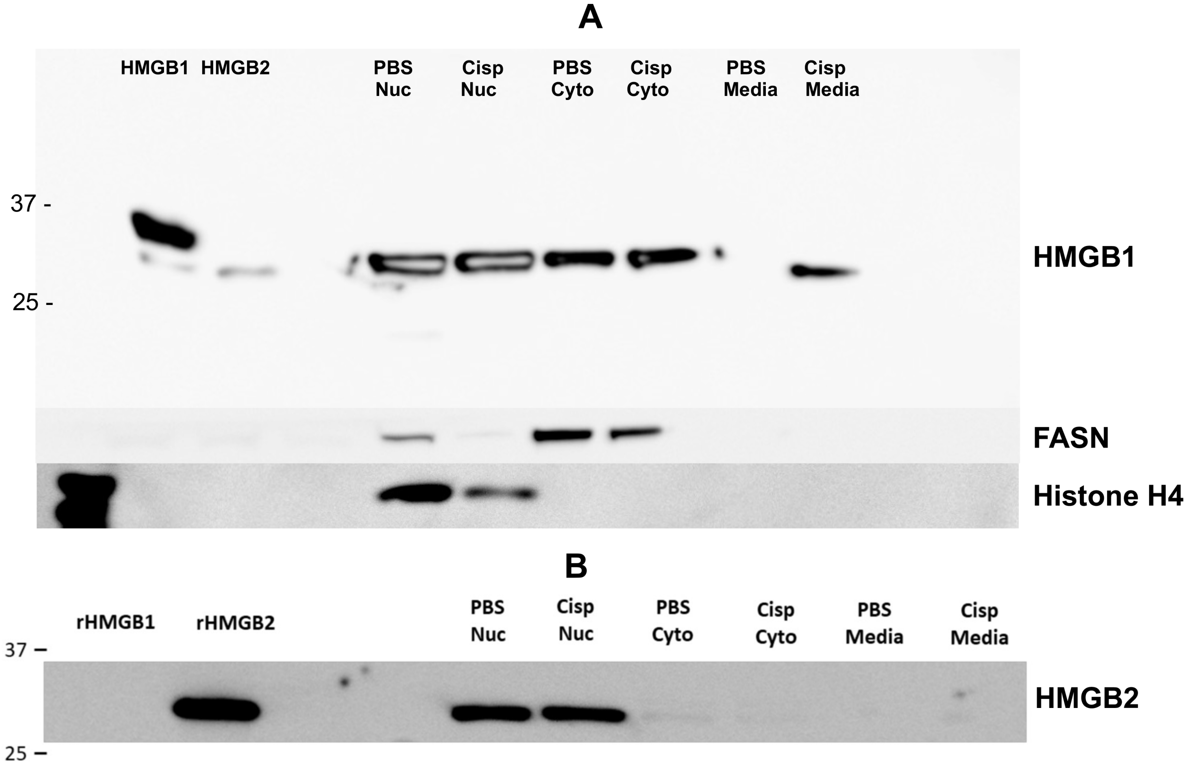

Figure S3. HMGB1 and HMGB2 in nuclear, cytosolic, and media fractions of A549 cells. (**A**) Representative immunoblots from biological replicates (n=3) for HMGB1 against recombinant HMGB1, recombinant HMGB2, nuclear extract, cytosolic extract, and extracellular media fractions from PBS and cisplatin (50 μM) treated A549 cells using an anti-HMGB1 rabbit pAb against the C-terminal acidic tail of HMGB1 tail of HMGB1. Immunoblots for FASN using an anti-FASN rabbit pAb and histone H4 using an anti-histone-4 rabbit pAb showed there was no contamination of the nuclear extract by cytosolic proteins or contamination of the cytosol by nuclear proteins. (**B**) Representative immunoblots from biological replicates (n=3) for HMGB2 against recombinant HMGB1, recombinant HMGB2, nuclear extract, cytosolic extract, and extracellular media fractions from PBS and cisplatin (50 μM) treated A549 cells using an anti-HMGB2 rabbit pAb raised against an N-terminal HMGB2 peptide of unspecified sequence. Protein plus protein dual-color standards were run on the same gel and visualized in black and white by an ImageQuant LAS 4000 camera. Images were superimposed and combined to mark molecular weights on the Western blot image.

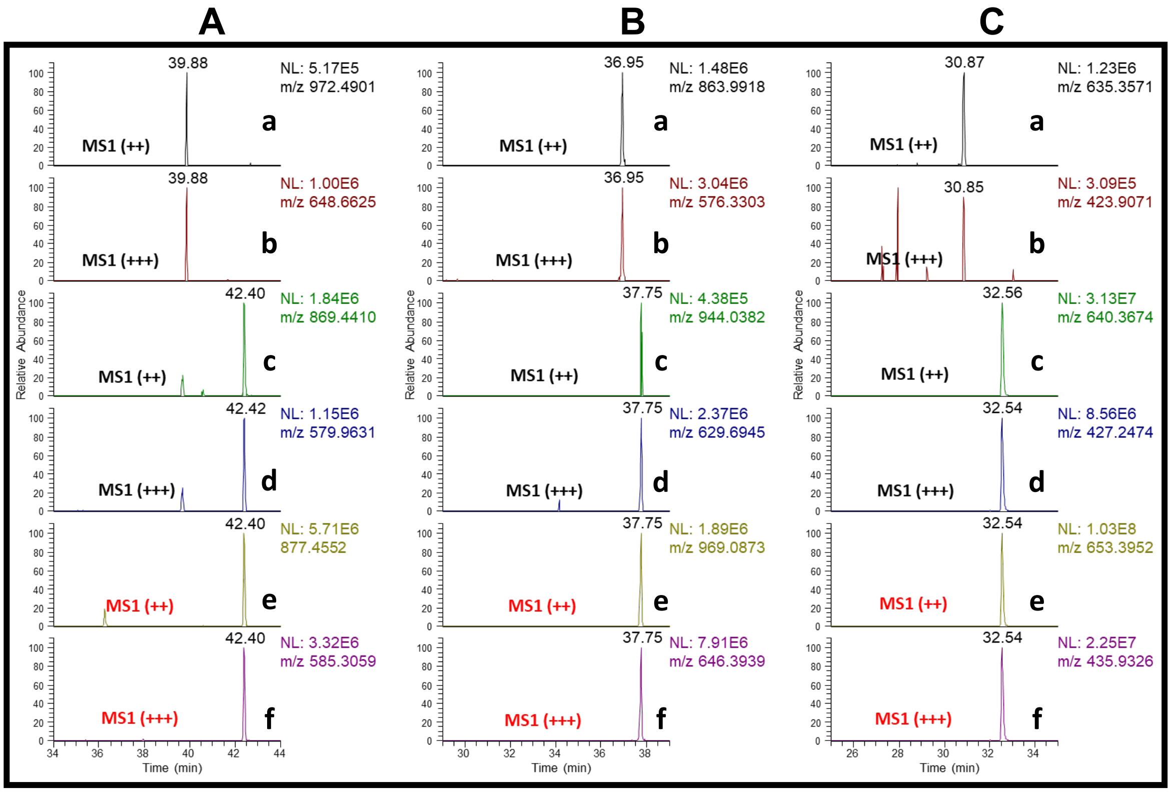

Figure S4. HMGB1 and HMGB2 in nuclear fraction of cisplatin treated A549 cells. Chromatograms from full scan spectra for corresponding light (black) and heavy (red) parent ion signals. **(A)** a-b HMGB2 Asp-N peptide D^{124}TAK(Ac*)K(Ac*)LGEMWSEQSAK^{139}; c-d HMGB1 Asp-N peptide D^{124}VAK(Ac*)K(Ac*)LGEMWNNTAA^{138}; e-f SILAC HMGB1 Asp-N peptide D^{124}VAK(Ac*)K(Ac*)LGEMWNNTAA^{138}. (**B**) a-b HMGB2 Asp-N peptide D^{140}K(Ac*)QPYEQK(Ac*)AAK(Ac*)LK^{152}(Ac*); c-d HMGB1 Asp-N peptide D^{139}DK(Ac*)QPYEK(Ac*)K(Ac*)AAK(Ac*)LK^{152}(Ac*); e-f SILAC HMGB1 Asp-N peptide D^{139}DK(Ac*)QPYEK(Ac*)K(Ac*)AAK(Ac*)LK^{152}(Ac*). (**C**) a-b HMGB2 Asp-N peptide D^{158I}AAYRAK(Ac*)GK(Ac*)S^{168}; c-d HMGB1 Asp-N peptide D^{158}IAAYRAK(Ac*)GK(Ac*)P^{168}; e-f SILAC HMGB1 Asp-N peptide D^{158}IAAYRAK(Ac*)GK(Ac*)P^{168}.

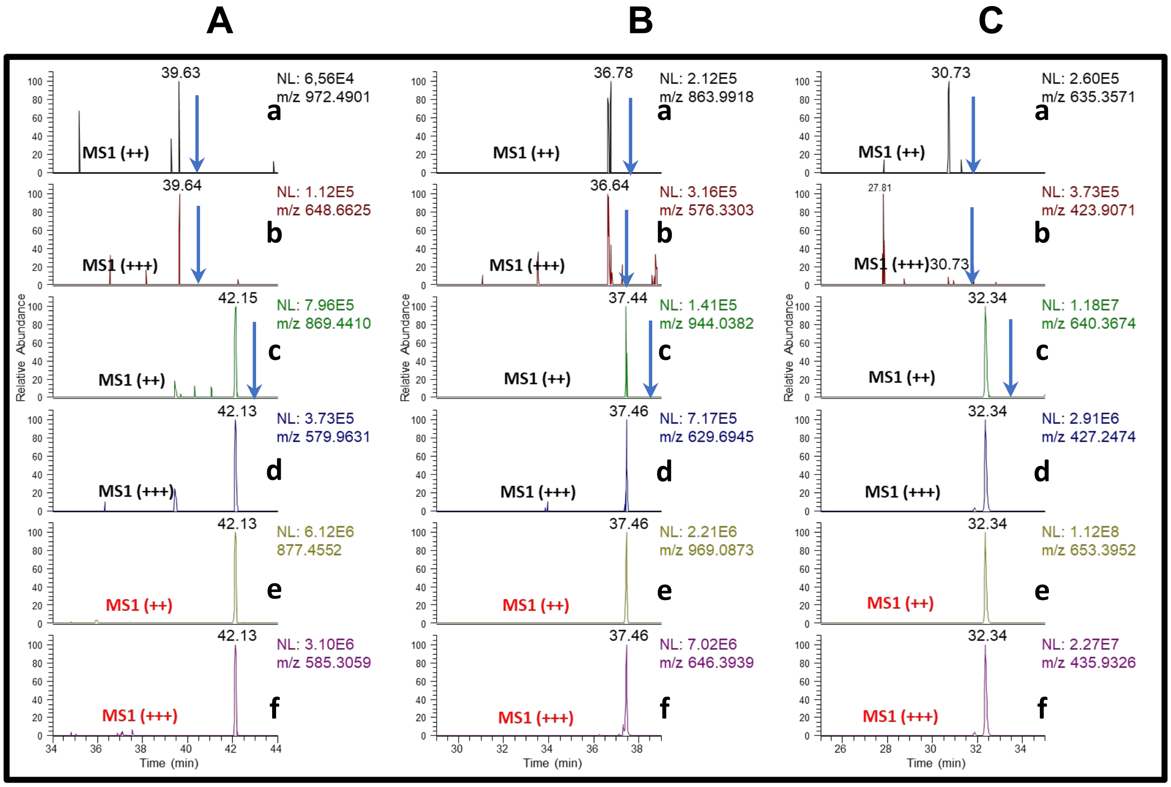

Figure S5. HMGB1 and HMGB2 in the cytosolic fraction of cisplatin treated A549 cells. Chromatograms from full scan spectra for corresponding light (black) and heavy (red) parent ion signals. **(A)** a-b HMGB2 Asp-N peptide D^{124}TAK(Ac*)K(Ac*)LGEMWSEQSAK^{139}; c-d HMGB1 Asp-N peptide D^{124}VAK(Ac*)K(Ac*)LGEMWNNTAA^{138}; e-f HMGB1 Asp-N peptide SILAC D^{124}VAK(Ac*)K(Ac*)LGEMWNNTAA^{138}. (**B**) HMGB2 Asp-N peptide a-b D^{140}K(Ac*)QPYEQK(Ac*)AAK(Ac*)LK^{152}(Ac*); c-d HMGB1 Asp-N peptide D^{139}DK(Ac*)QPYEK(Ac*)K(Ac*)AAK(Ac*)LK^{152}(Ac*); e-f SILAC HMGB1 Asp-N peptide D^{139}DK(Ac*)QPYEK(Ac*)K(Ac*)AAK(Ac*)LK^{152}(Ac*). (**C**) a-b HMGB2 Asp-N peptide D^{158I}AAYRAK(Ac*)GK(Ac*)S^{168}; c-d HMGB1 Asp-N peptide D^{158}IAAYRAK(Ac*)GK(Ac*)P^{168}; e-f SILAC HMGB1 Asp-N peptide D^{158}IAAYRAK(Ac*)GK(Ac*)P^{168}. Blue arrows indicate missing signals for HMGB2 that were observed in the nuclear fraction.

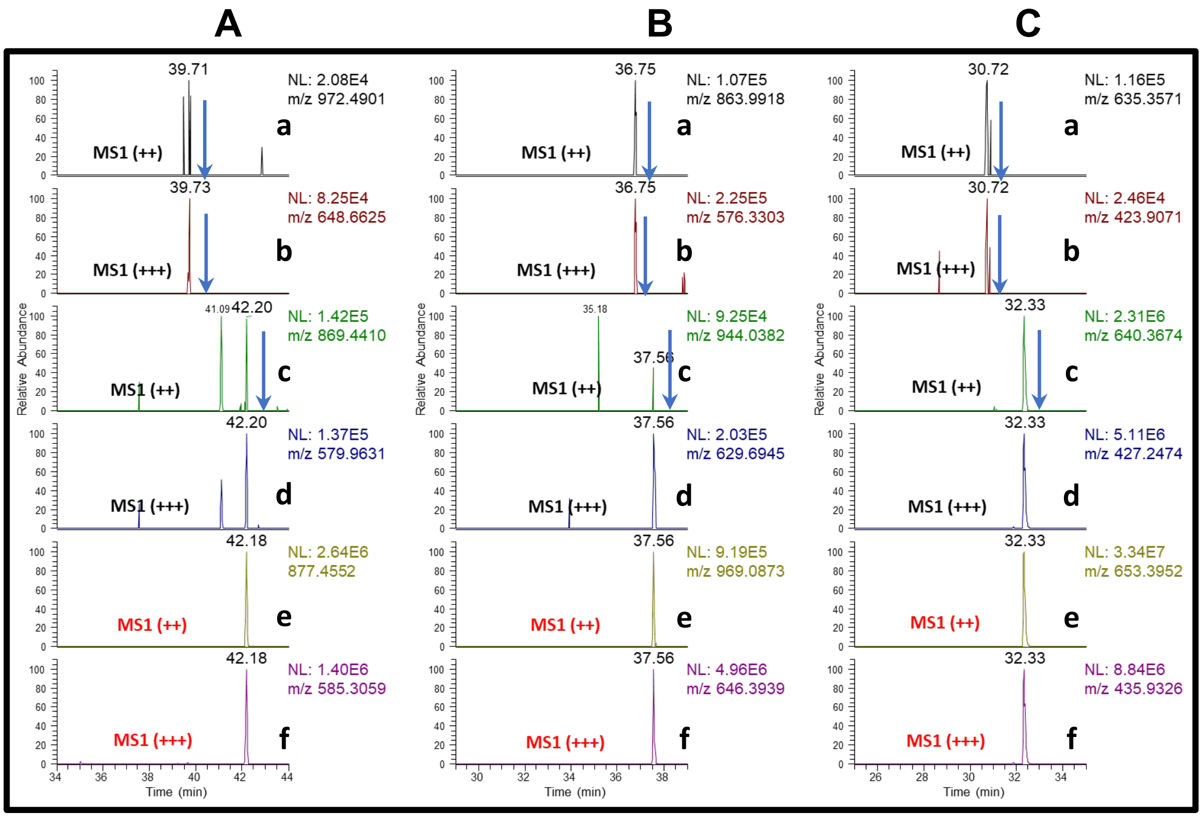

Figure S6. HMGB1 and HMGB2 in media from cisplatin treated A549 cells. Chromatograms from full scan spectra for corresponding light (black) and heavy (red) parent ion signals. **(A)** a-b HMGB2 Asp-N peptide D^{124}TAK(Ac*)K(Ac*)LGEMWSEQSAK^{139}; c-d HMGB1 Asp-N peptide D^{124}VAK(Ac*)K(Ac*)LGEMWNNTAA^{138}; e-f SILAC HMGB1 Asp-N peptide D^{124}VAK(Ac*)K(Ac*)LGEMWNNTAA^{138}. (**B**) a-b HMGB2 Asp-N peptide D^{140}K(Ac*)QPYEQK(Ac*)AAK(Ac*)LK^{152}(Ac*); c-d HMGB1 Asp-N peptide D^{139}DK(Ac*)QPYEK(Ac*)K(Ac*)AAK(Ac*)LK^{152}(Ac*); e-f SILAC HMGB1 Asp-N peptide D^{139}DK(Ac*)QPYEK(Ac*)K(Ac*)AAK(Ac*)LK^{152}(Ac*). (**C**) a-b HMGB2 Asp-N peptide D^{158I}AAYRAK(Ac*)GK(Ac*)S^{168}; c-d HMGB1 Asp-N peptide D^{158}IAAYRAK(Ac*)GK(Ac*)P^{168}; e-f SILAC HMGB1 Asp-N peptide D^{158}IAAYRAK(Ac*)GK(Ac*)P^{168}. Blue arrows indicate missing signals for HMGB2 that were observed in the nuclear fraction.

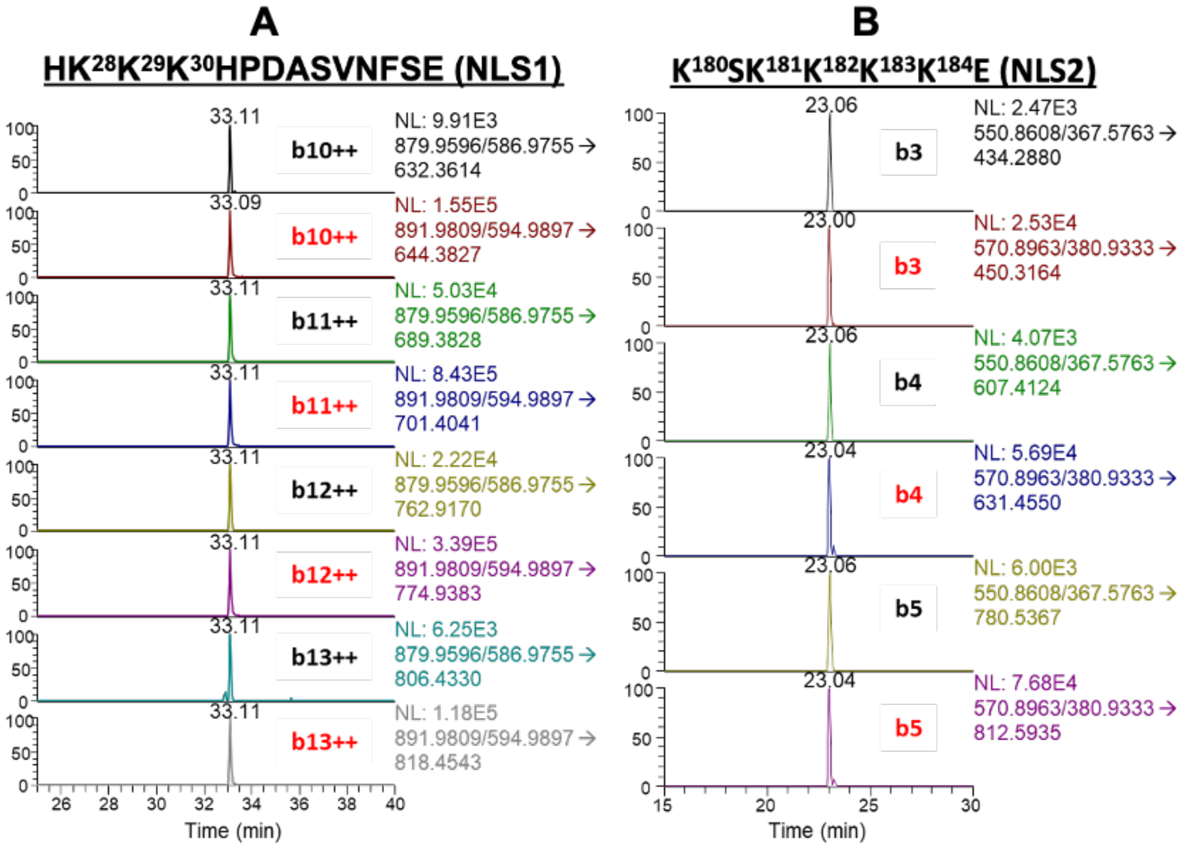

Figure S7. NLS1 and NLS2 transitions of unacetylated HMGB1 secreted with cisplatin treatment. Lysine-containing peptides from cisplatin-treated A549 media were analyzed for acetylation by nanoLC-PRM. Chromatograms depict light (black) and heavy (red) transitions with *C*D3 acetylation. **(A)** PRM/HRMS chromatograms for NLS1 peptide HK^{28}K^{29}K^{30}HPDASVNFSE depicting light and heavy NLS1 peptide transitions. **(B)** NanoLC-PRM/HRMS chromatograms for NLS2 peptide K^{180}SK^{181}K^{182}K^{183}K^{184} depicting light and heavy NLS2 peptide transitions.

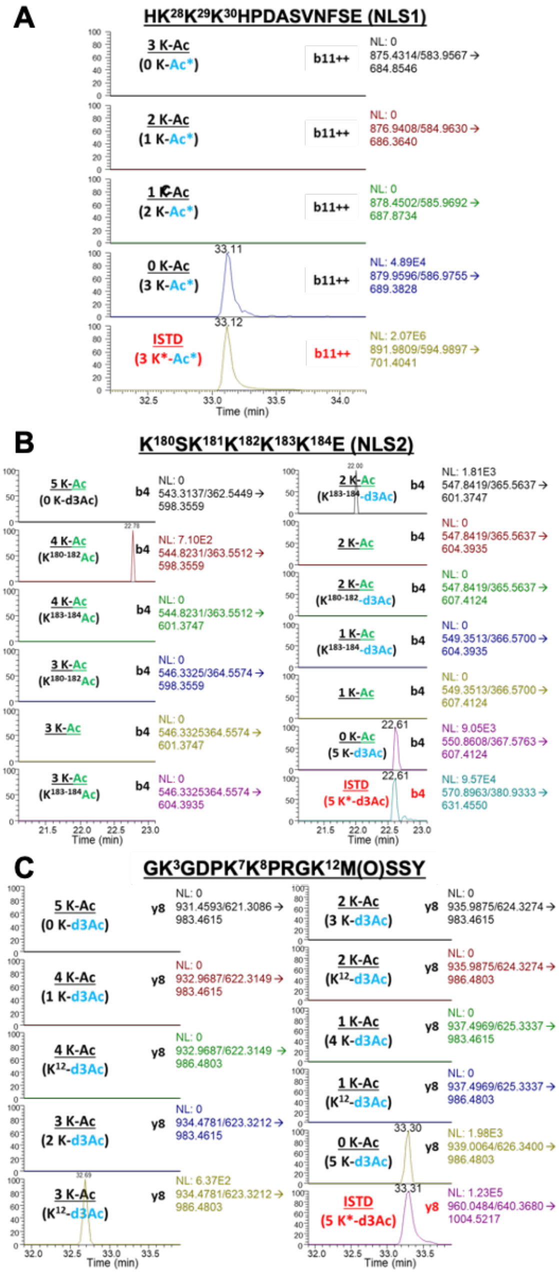

Figure S8. DMSO induces secretion of unacetylated HMGB1. Lysine-containing peptides from DMSO-treated A549 media were analyzed for acetylation by PRM. PRM/HRMS chromatograms depict light (black) and heavy (red) transitions with C*D*3 or endogenous acetylation. **(A)** PRM/HRMS chromatograms for NLS1 peptide HK^{28}K^{29}K^{30}HPDASVNFSE comparing b11 transitions for triply (first panel), doubly (second panel), and singly (third panel) acetylated NLS1 compared to unacetylated NLS1 peptide (fourth panel) and heavy NLS1 peptide (fifth panel). (**B)** PRM/HRMS chromatograms for NLS2 peptide K^{180}SK^{181}K^{182}K^{183}K^{184} comparing y8 transitions of different possible acetylation states. **(C)** PRM/HRMS chromatograms for N-terminal peptide GK^{3}GDPK^{7}K^{8}PRGK^{12}M(O)SSY comparing b4 transitions of different possible acetylation states.

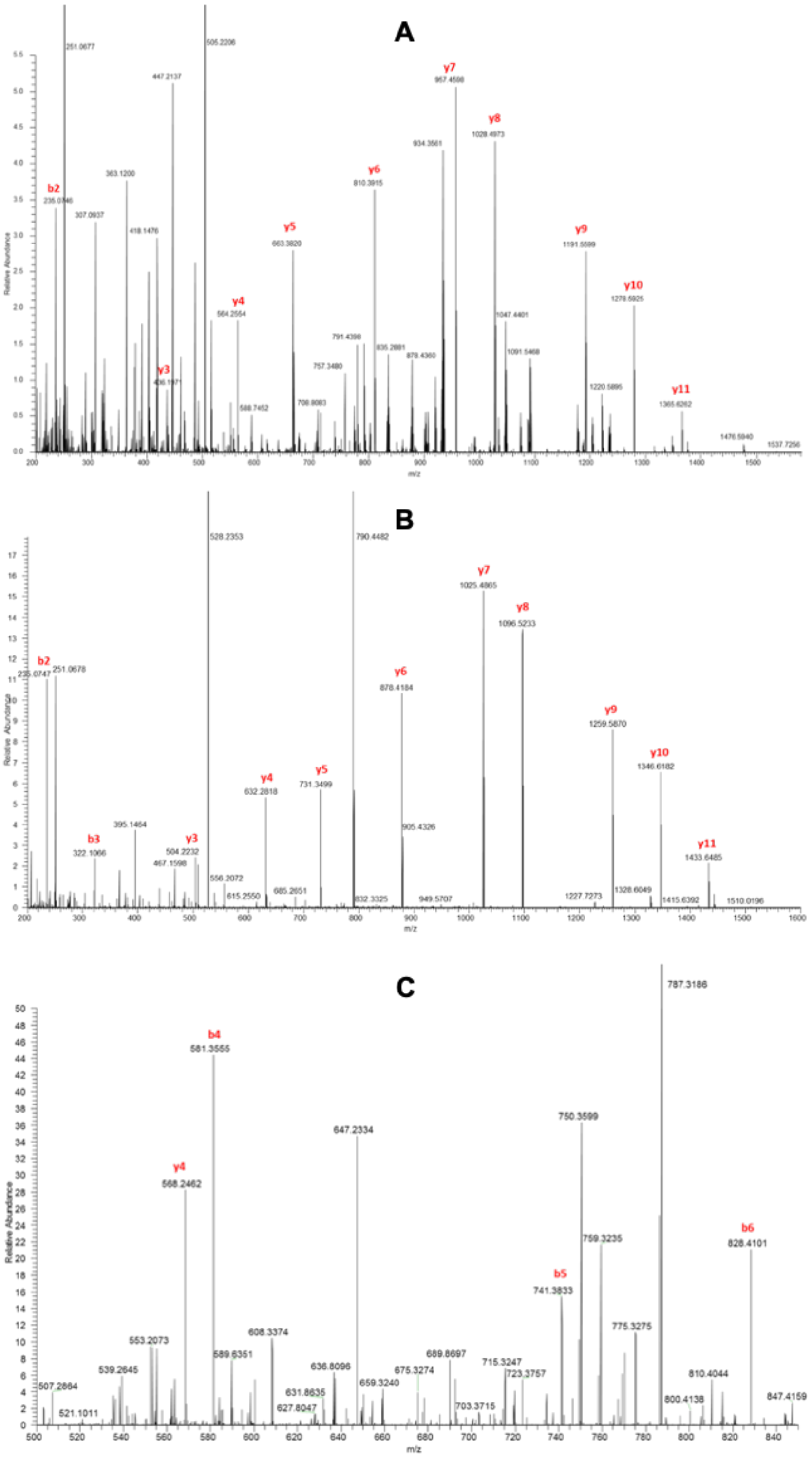

Figure S9. Assigned mass spectra in cysteine-containing peptides in HMGB1 secreted with cisplatin treatment I. M(O)SSYAFFQTC[23]R and FSK(d3Ac)K(d3Ac)C[45]SE. **(A***)* M(O)SSYAFFQTC[23]R – CAM. **(B)** M(O)SSYAFFQTC[23]R – NEM. **(C)** FSK(d3Ac)K(d3Ac)C[45]SE – CAM.

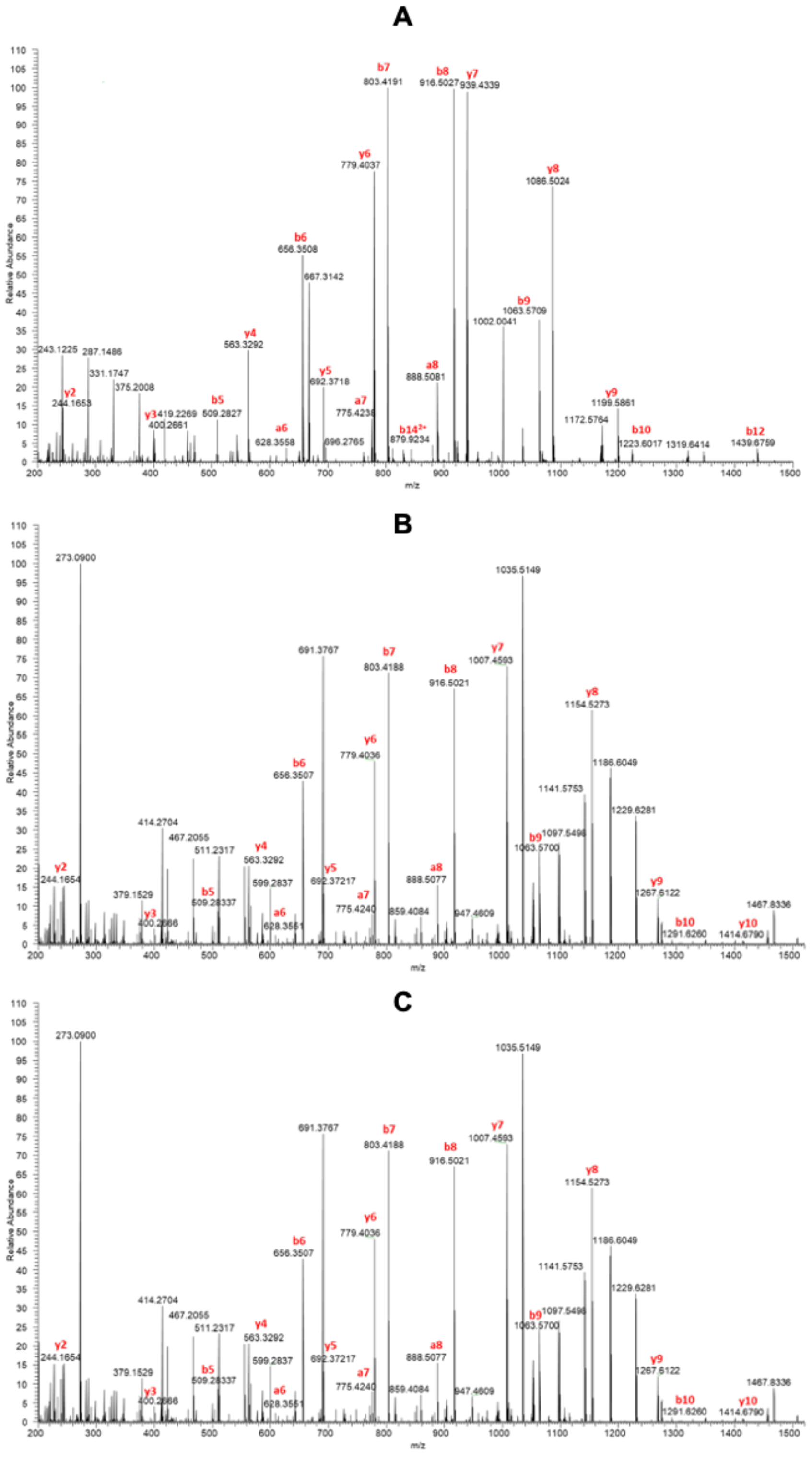

Figure S10. Assigned mass spectra in cysteine-containing peptides in HMGB1 secreted with cisplatin treatment II. FSK(d3Ac)K(d3Ac)C^{45}SE and RPPSAFFLFC^{106}SEYRPK. **(A)** FSK(d3Ac)K(d3Ac)C^{45}SE – NEM. **(B)** RPPSAFFLFC^{106}SEYRPK – CAM. **(C)** RPPSAFFLFC^{106}SEYRPK – NEM.

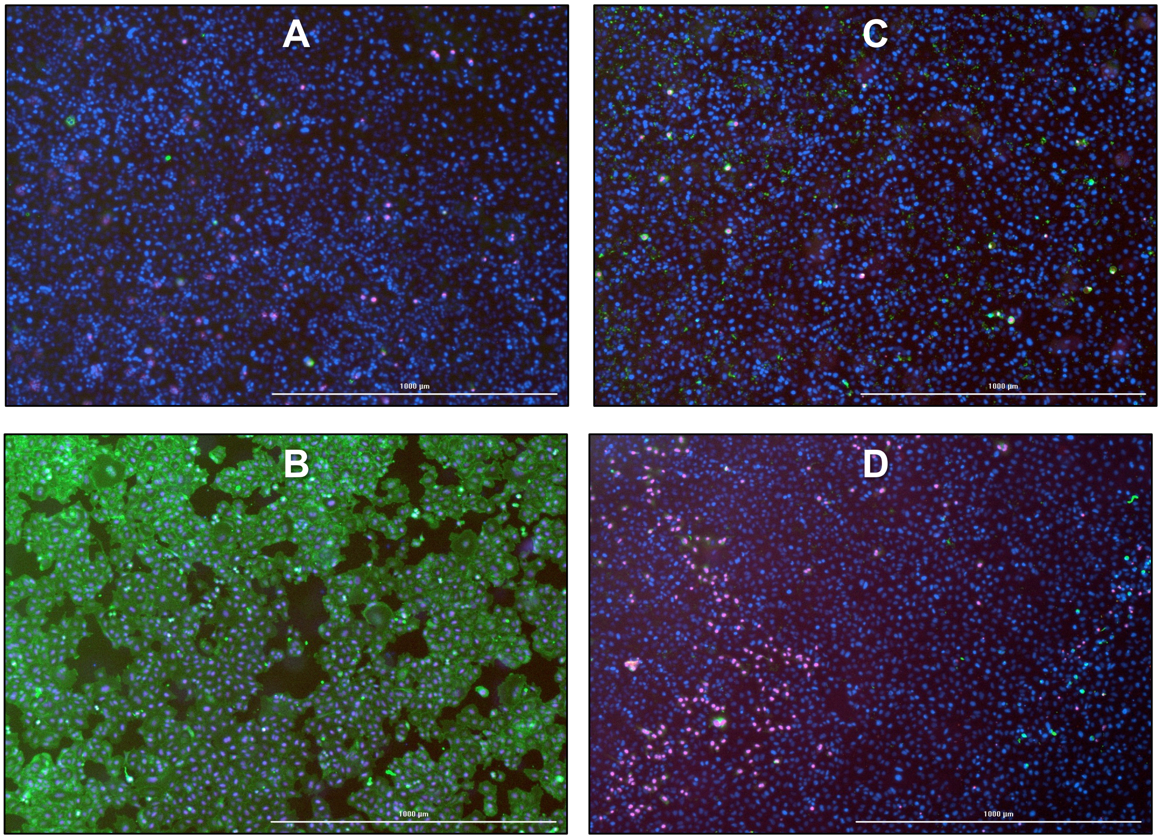

Figure S11. Cisplatin-mediated secretion of HMGB1 fromA549 cells is not dependent on necrosis. Representative overlayed fluorescent microscopy analysis of DNA stain Hoechst (blue) and apoptosis-specific stain pSIVA (green) from biological replicates (n=3) of A549 cells after 24-h treatment with: (**A**) PBS (control). (**B**) 2 mM DMSO. (**C**) 20 µM cisplatin. (**D**)100 µM cisplatin.

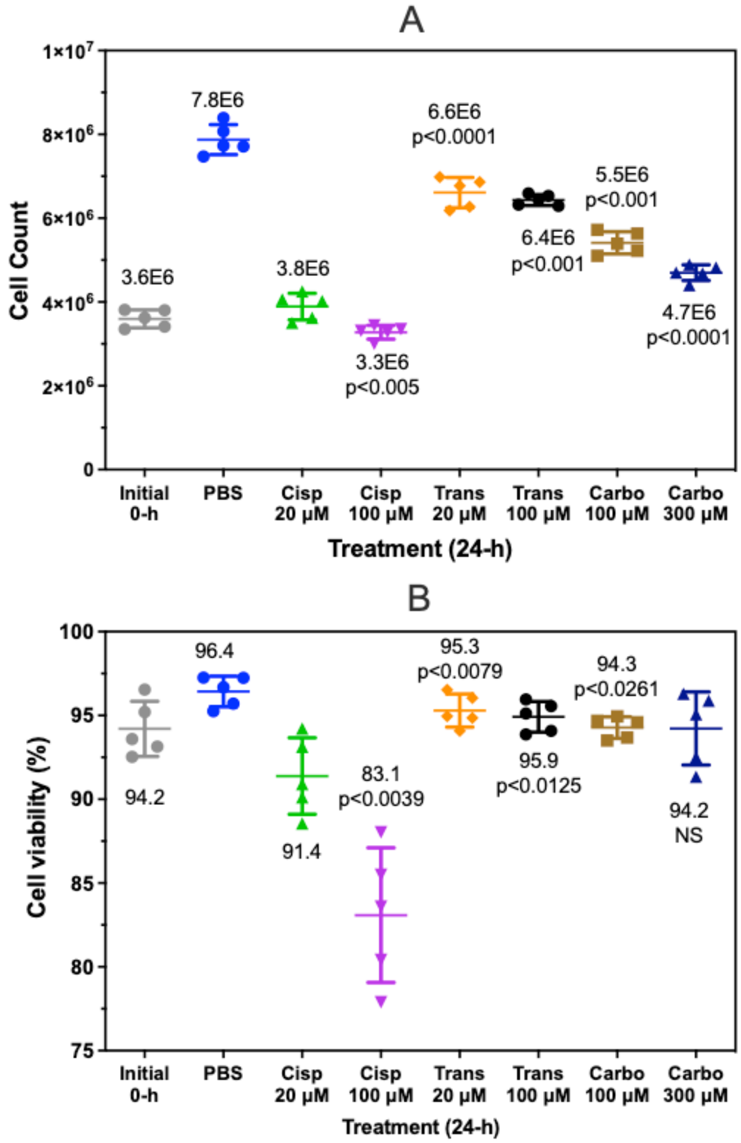

Figure S12. Cisplatin attenuates A549 cell growth and decreases viability more than other platinum drug analogs. Trypan blue exclusion assay on biological replicates (n = 5) comparing platinum drug analogs. Cisplatin (20 µM and 100 µM), transplatin (20 µM and 100 µM) and carboplatin (100 µM and 300 µM) compared after 24-h treatment with respect to PBS vehicle control. **(A)** Viable cell count after 24-h. **(B)** Cell viability (%) after 24-h. Student's t statistical test was used to compare cisplatin (20 M) with other treatments.
