# Supplementary material for: Cisplatin Dependent Secretion of Immunomodulatory High Mobility Group Box 1 (HMGB1) Protein from Lung Cancer Cells
Source: Biomolecules. 2023 Aug 31;13(9):1335. doi: 10.3390/biom13091335 (PMC10526420; doi:10.3390/biom13091335)
Supplement: Supplementary file 1 [file biomolecules-13-01335-s001.zip › biomolecules-2527825-Figure S13.pdf]

Full size immunoblots

A

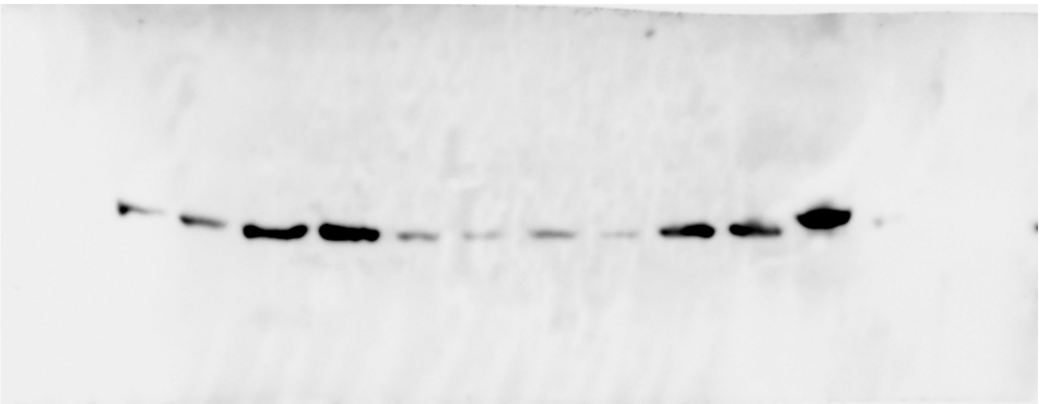

B

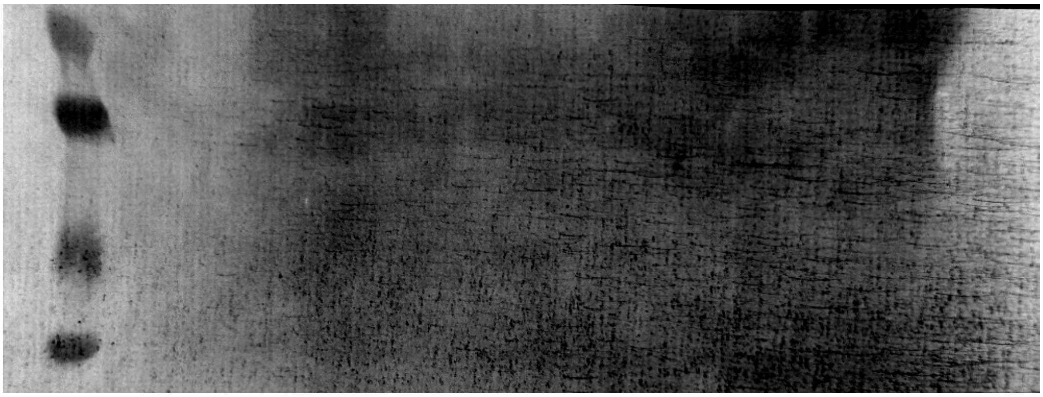

Figure 2A. Cisplatin induces HMGB1 secretion from lung cancer cells. (A) Full immunoblots from anti-HMGB1 pAb following 24-h incubation A549 cells with cisplatin (Cisp, 20 μM), Cisp (100 μM, transplatin (Transp, 20 μM), Transp (100 μM), carboplatin (Carbop, 100 μM) and Carbop (300 μM) and representative HMGB1 immunoblots from untreated malignant mesothelioma cell lines REN and EMMESO. (**B**) Protein plus protein dual-color standards were run on the same gel and visualized in black and white by an ImageQuant LAS 4000 camera. Images were superimposed and combined to mark molecular weights on the Western blot image.

A

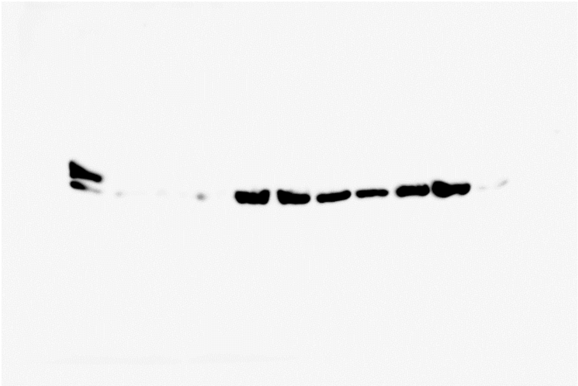

B

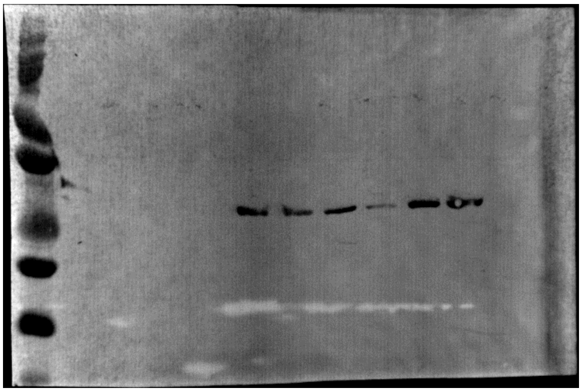

Figure 6A. Cisplatin mediated high mobility group box 1 (HMGB1) secretion is regulated by nuclear exportin 1 (XPO1). (**A**) Anti-HMGB1 pAb after 50 µM cisplatin and Olaparib (50 µM). (**B**) Protein plus protein dual-color standards were run on the same gel and visualized in black and white by an ImageQuant LAS 4000 camera. Images were superimposed and combined to mark molecular weights on the Western blot image.

A

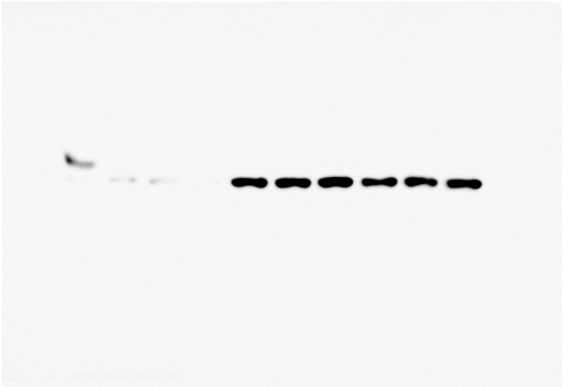

B

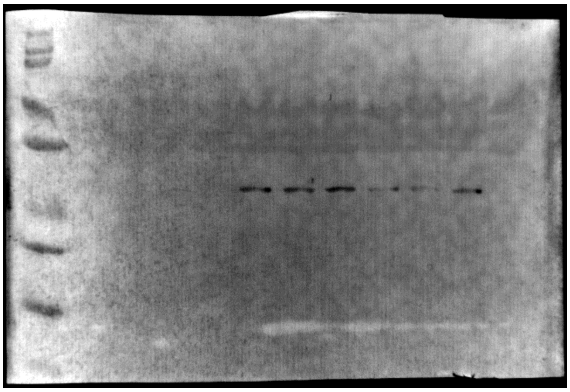

Figure 6B. Cisplatin mediated high mobility group box 1 (HMGB1) secretion is regulated by nuclear exportin 1 (XPO1). (**A**) Anti-HMGB1 pAb after 50 µM cisplatin and GO-6983 (120 nM). (**B**) Protein plus protein dual-color standards were run on the same gel and visualized in black and white by an ImageQuant LAS 4000 camera. Images were superimposed and combined to mark molecular weights on the Western blot image.

A

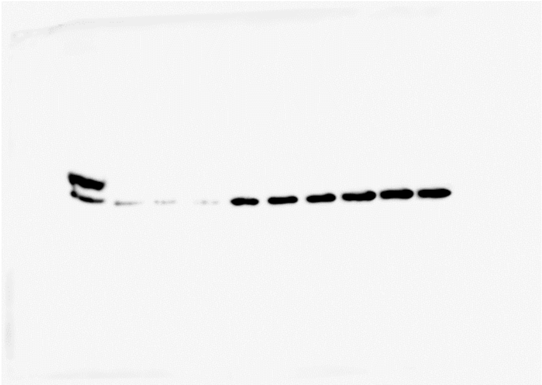

B

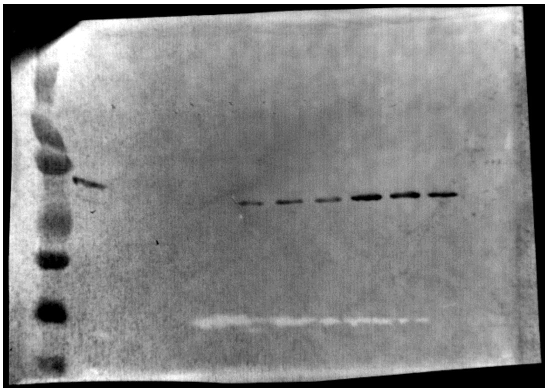

Figure 6C. Cisplatin mediated high mobility group box 1 (HMGB1) secretion is regulated by nuclear exportin 1 (XPO1). (A) Anti-HMGB1 pAb after 50 µM cisplatin and Rottlerin (100 µM). (**B**) Protein plus protein dual-color standards were run on the same gel and visualized in black and white by an ImageQuant LAS 4000 camera. Images were superimposed and combined to mark molecular weights on the Western blot image.

A

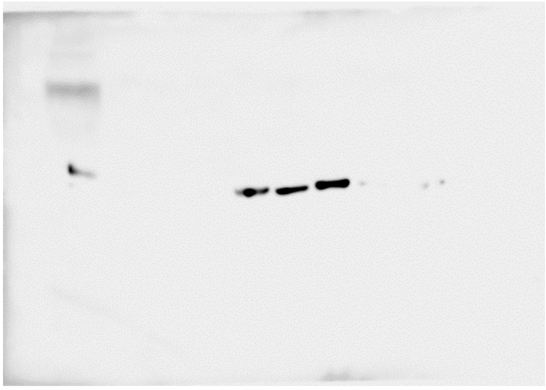

B

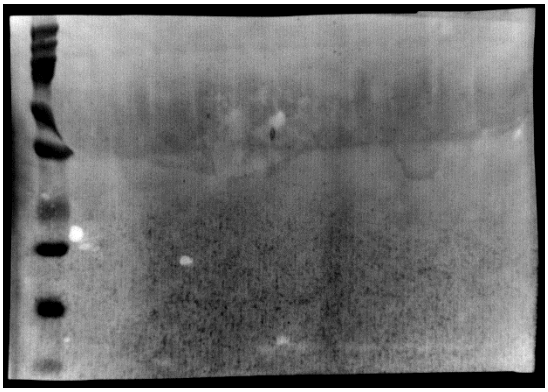

Figure 6D. Cisplatin mediated high mobility group box 1 (HMGB1) secretion is regulated by nuclear exportin 1 (XPO1). (A) Anti-HMGB1 pAb after 50 μM cisplatin and Leptomycin B (4.5 nM). (**B**) Protein plus protein dual-color standards were run on the same gel and visualized in black and white by an ImageQuant LAS 4000 camera. Images were superimposed and combined to mark molecular weights on the Western blot image.

A

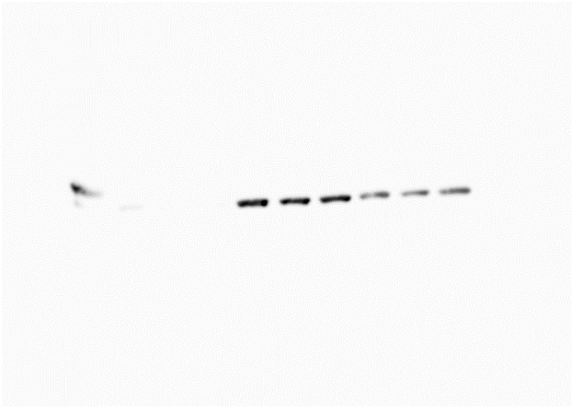

B

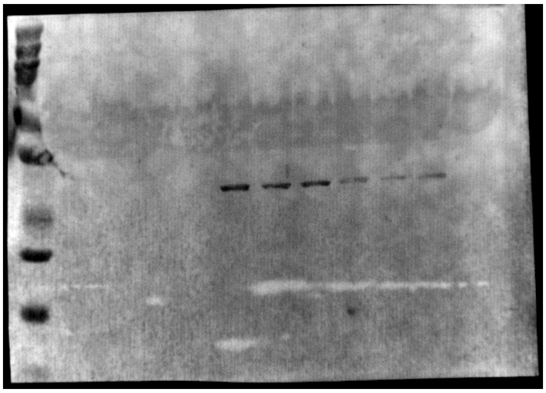

Figure 6E. Cisplatin mediated high mobility group box 1 (HMGB1) secretion is regulated by nuclear exportin 1 (XPO1). (A) Anti-HMGB1 pAb after 50 μM cisplatin and KPT-330 (75 nM). (**B**) Protein plus protein dual-color standards were run on the same gel and visualized in black and white by an ImageQuant LAS 4000 camera. Images were superimposed and combined to mark molecular weights on the Western blot image.

A

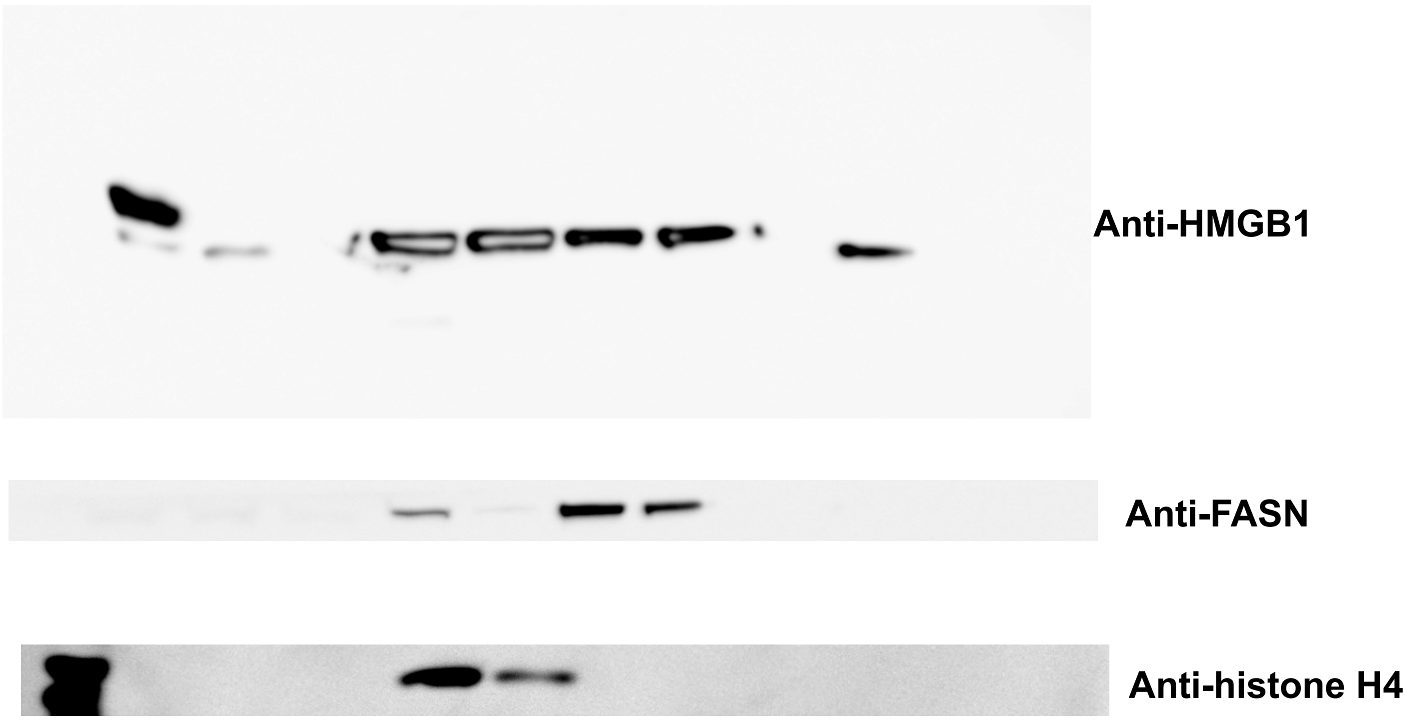

B

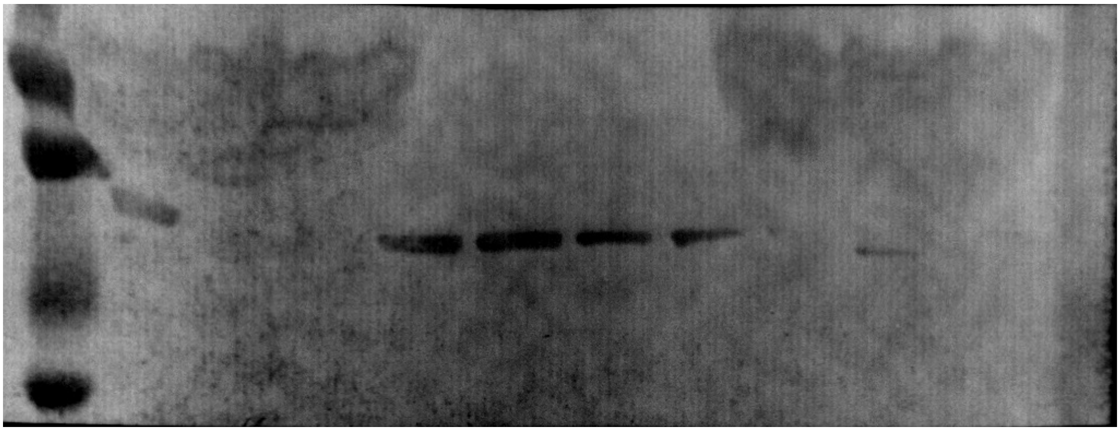

Figure S3A. HMGB1 and HMGB2 in nuclear, cytosolic, and media fractions of A549 cells. (**A**) Representative full size immunoblots of HMGB1, FASN and histone H4. Cytosolic and nuclear fraction were tested with immunoblotting for FASN and histone-4 rabbit pAbs, respectively. This process followed standard immunoblotting protocol for loading controls to ensure that any observed differences in HMGB1 or HMGB2 signals were due to differences in protein expression levels rather than errors in subcellular fraction preparation, gel loading, or protein transfer. Following SDS-PAGE transfer to nitrocellulose membrane, the membrane was cut horizontally above 75 kDa protein ladder marker up to right above 250 kDa protein ladder marker for eventual incubation with anti-FASN rabbit pAb. Similarly, the membrane was cut horizontally right below 20 kDa protein ladder marker down to right below 10 kDa protein ladder marker for eventual incubation with anti-H4 rabbit pAb. (**B**) Protein plus protein dual-color standards were run on the same gel and visualized in black and white by an ImageQuant LAS 4000 camera. Images were superimposed and combined to mark molecular weights on the Western blot image.

A

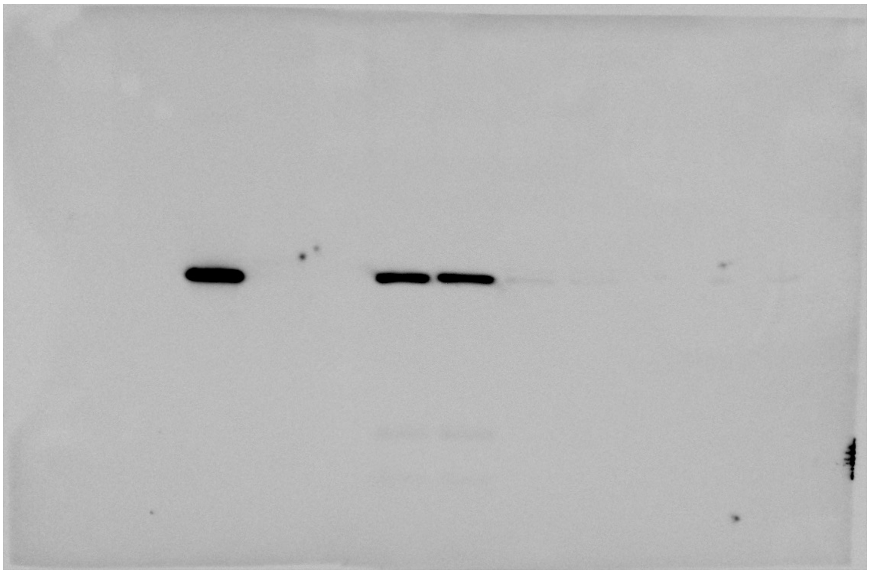

B

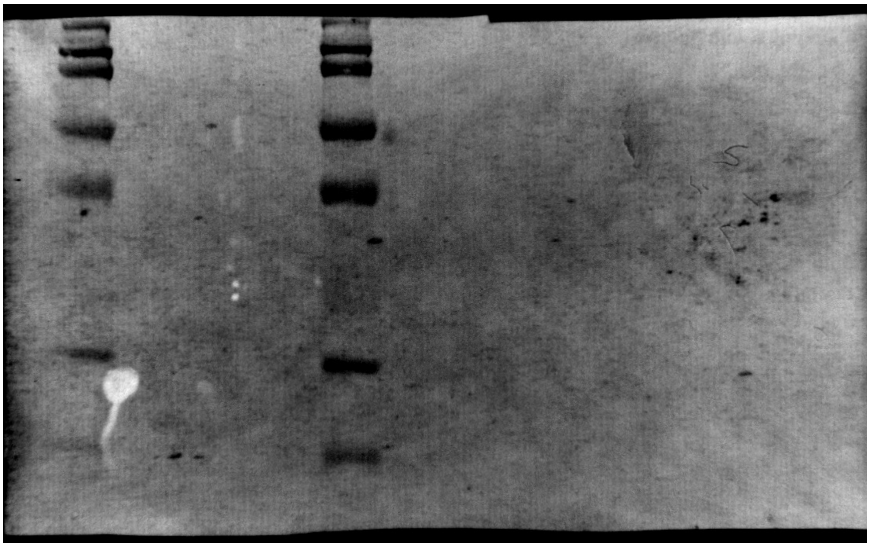

Figure S3B. HMGB1 and HMGB2 in nuclear, cytosolic, and media fractions of A549 cells. (**A**) Representative full size immunoblots. (**B**) Protein plus protein dual-color standards were run on the same gel and visualized in black and white by an ImageQuant LAS 4000 camera. Images were superimposed and combined to mark molecular weights on the Western blot image.
